# Effects of Soil Oxygen Conditions and Soil pH on Remediation of DDT-contaminated Soil by Laccase from White Rot Fungi

**DOI:** 10.3390/ijerph7041612

**Published:** 2010-04-07

**Authors:** Yuechun Zhao, Xiaoyun Yi

**Affiliations:** 1 Department of Applied Chemistry, South China Agricultural University, Guangzhou 510642, China; 2 College of Environmental Science and Engineering, South China University of Science & Technology, Guangzhou 510640, China; E-Mail: xyyi@scut.edu.cn

**Keywords:** laccase, DDT-contaminated soil, remediation, soil oxygen conditions, soil pH

## Abstract

High residues of DDT in agricultural soils are of concern because they present serious threats to food security and human health. This article focuses on remediation of DDT-contaminated soil using laccase under different soil oxygen and soil pH conditions. The laboratory experiment results showed significant effects of soil oxygen conditions and soil pH on remediation of DDT-contaminated soil by laccase at the end of a 25-d incubation period. This study found the positive correlation between the concentration of oxygen in soil and the degradation of DDT by laccase. The residue of DDTs in soil under the atmosphere of oxygen decreased by 28.1% compared with the atmosphere of nitrogen at the end of the incubation with laccase. A similar pattern was observed in the remediation of DDT-contaminated soil by laccase under different flooding conditions, the higher the concentrations of oxygen in soil, the lower the residues of four DDT components and DDTs in soils. The residue of DDTs in the nonflooding soil declined by 16.7% compared to the flooded soil at the end of the incubation. The residues of DDTs in soils treated with laccase were lower in the pH range 2.5–4.5.

## 1. Introduction

1,1,1-Trichloro-2,2-bis (4-chlorophenyl) ethane (DDT) was a widely used organochlorine pesticide with high toxicity and residual levels. In 2001 the Governing Council of the United Nations Environment Programme issued a treaty to eliminate or restrict the production and use of persistent organic pollutants (POPs). Twelve offenders were listed and DDT is among them. In U.S. and some other countries a ban on DDT took effect earlier [1]. Although the use of DDT for wide agricultural use has been banned in China since 1983, a large amount of DDT still remains in soils. In some areas the DDT concentration found in soil markedly exceeds the level set by the national soil quality standards (GB/T 18407-2001). For example, the concentration of DDT exceeded the national soil quality standards in the agricultural soils of North of Zhejiang Province [2]. In Tianjin region, the concentration of p,p′-DDT and p,p′-DDE in the soils was 27.5 and 18.8 ng/g, respectively [3]. Soil pollutants may adversely affect agricultural products. The rate of detection DDT in some vegetables produced in a Nanjing suburb was up to 100% [4]. Therefore, remediation of the DDT-contaminated soils must be conducted to ensure the agricultural products safety and human health.

Recently, some physical, chemical and biological approaches have been researched to clean up DDT in the soils. For example, the physical approaches of bioventing and thermal treatment [5] and chemical remediation of leaching with surfactants [6], oxidation [7], dechlorination by metallic reduction [8,9] and light degradation [10]. However, remediation using physical approaches is expensive, and chemical approaches adversely affect the soils physical-chemical properties, causing secondary pollution. Bioremediation methods, mainly phytoremediation [11–14] and microbial bioremediation [15–17], have been utilized to remove DDT from contaminated soils. Enzymatic remediation is a rapid and efficient method of removing pesticide residue from the environment [18]. Laccase (EC 1.10.3.2) can catalyze the oxidation of organic compounds [19], and has been widely used to degrade pollutants from environment [20–22]. However, the environmental conditions of soil are complex. Enzymatic activity may be reduced or eliminated by degradation of proteases, adsorption on soil colloids and extreme acidity or alkalinity *etc.* [23]. In this study, the effects of different soil oxygen conditions and different soil pH on remediation of DDT-contaminated soil by laccase were investigated in laboratory batch experiments.

## 2. Materials and Methods

### 2.1. Materials

White rot fungi (*Panus conchatus*) used for the production of laccase were purchased from Guangzhou Chemical Company Ltd of the Chinese Academy of Sciences (Guangzhou, China). Standards of the four components of DDT (p,p′-DDE, p,p′-DDT, p,p′-DDD and p,p′-DDT) were purchased from Supelco (Park Bellefonte, PA, USA). The experimental soil was woodland soil that was obtained from the arboretum of South China Agricultural University. The basic physical and chemical properties of the soil after air drying were measured. The soil texture was silty soil, the soil pH was 5.87, organic matter was 9.6 g/ kg, and total iron was 39 mg/kg. DDTs in this study stands for the total sum of p,p′-DDE, o,p′-DDT, p,p′-DDD and p,p′-DDT in each sample. DDT was not detected in these soils (the detection limit will be described later).

### 2.2. Preparation of Laccase and Enzymatic Activity Measurement

White rot fungi was cultivated for nine days on solid agar medium plates. The solid agar medium employed in the experiment contained (g/L): KCl, 0.1; MgSO_4_·7H_2_O, 0.3; KH_2_PO_4_, 1; NaNO_3_, 2.5; glucose, 20; agar, 20; potato flour, 20 and the following micronutrients (mg/L): FeCl_3_·6H_2_O, 10; CaCl_2_·2H_2_O, 10; VB1, 10. Final pH was 6.0. A mycelial mat of appropriate growth phase was cut from the solid agar medium plates by a 10 mm hole diameter puncher and blended with 50 mL potato liquid medium for precultures. The liquid medium in the experiment was made with 1,000 mL of potato extract and the following components (g/L): MgSO_4_·7H_2_O, 1.5; KH_2_PO_4_, 3; yeast extract, 5; glucose, 20; VB1, 0.01. Precultures were prepared for four days at 130 rpm and 28 °C. Crude laccase preparation was carried out in 1 L Erlenmeyer flasks with the same potato liquid medium for nine days at 130 rpm and 28 °C. The crude laccase was filtered and centrifuged. The supernatant was brought to 20% saturation ((NH_4_) _2_SO_4_, overnight at 4 °C) and then centrifuged. The resulting supernatant was brought to 80% saturation ((NH_4_) _2_SO_4_, overnight at 4 °C) and then centrifuged. The supernatant was loaded onto a Sephadex G-75 column equilibrated with 0.02 mol/L sodium acetate buffer (pH 4.6). Proteins were eluted with 0–0.5 mol/L NaCl at a flow rate of 0.5 mL/min. Eluted fractions containing laccase activity were pooled and kept (4 °C). Laccase activity was determined by monitoring the oxidation of ABTS [2,2′-azino-bis(3-ethylbenzthiazoline-6-sulphonic acid)] at 420 nm. 1 U of activity was defined as the amount of enzyme able to oxidize 1 μmol ABTS per min. Regardless of the initial amount of laccase added, the activity of laccase reduced gradually during a 20-d incubation under different conditions. Laccase lost 100% of the initial activity in any of the soil microcosms after a 20-d exposure to soil.

### 2.3. Preparation of DDT-contaminated Soils

Commercial DDT (0.0547 g) was dissolved in 100 mL acetone in a volumetric flask. The DDT solution was kept with a volumetric flask and stored at 4 °C for use; 5 mL of 0.547 mg/mL DDT solution was added into 50 g soil passed through a 2-mm sieve, stirred well and air-dried. Then the dried sample was put into 450 g soil, sieved at 2 mm, and mixed well. This was the experimental DDT-contaminated soil sample. The soil sample was kept at room temperature for four weeks, after that remediation experiments were carried out to assess the remedial potential of laccase. Throughout the experiment, the water content of all soil samples was kept at approximately 15%. The initial contents of DDT components and DDTs in the soil samples were 0.351 mg/kg (p,p′- DDE), 0.775 mg/kg (o,p′-DDT), 1.403 mg/kg (p,p′-DDD), 2.334 mg/kg (p,p′-DDT) and 4.863 mg/kg (DDTs), respectively.

### 2.4. Enzymatic Remediation of DDT-contaminated Soil

To study the effects of different atmospheres on remediation of DDT-contaminated soil by laccase experiments were conducted under three atmospheres (air, oxygen and nitrogen). To examine the effects of different flooding conditions on remediation of DDT-contaminated soil by laccase the water content in soil samples of nonflooding was kept at approximately 15%. The water content in intermittent flooding soil samples was kept at approximately 15% during the first 12 days, and then the soil samples were flooded until the end of experiment. The flooding soil samples were flooded for 25 days. To create flooding condition of the soils, distilled water level in the beaker reached 4 cm. The laccase was split into two portions and added to the soil on day 1 and day 13 of the incubation experiment.

To study the effects of different soil pH on remediation of DDT-contaminated soil by laccase the experimental soil was leached for 24 h using phosphate buffer solution (pH 2.5–6.5). pH of the soils were 2.5, 3.5, 4.5, 5.5 and 6.5, respectively.

For each experiments (atmospheres, flooding conditions, pH) one control experiment without laccase (0 U/g soil) and one degradation experiment with laccase (6 U/g soil) were performed. Each experiment used 15-g soil. All experiments were conducted in beakers, and replicated three times. Soil samples were analyzed at 25 d.

### 2.5. Sample Pre-Treatment

The preparation of sample and the measurement of DDT concentration were conducted according to the standards described in national standards of P. R. China (GB/T14550-93) set for Soil Quality-Determination of BHC and DDT-Gas chromatography. Briefly, 10 g of soils were placed in a Soxhlet extraction apparatus and immersed with 100 mL petroleum ether-methanol (1:1) solution for 10 h, then extracted for 6 h. The extract liquid was transferred to a separating funnel and 10% aqueous sodium sulfate was added. After 1 min of shaking, the mixture stratified into two liquid phases on standing. The upper layer petroleum ether was separated and treated with concentrated sulfuric acid with a volume equivalent to 10% of the petroleum ether solution. The mixture of petroleum ether and sulfuric acid was shaken 3–4 times until both layers became colorless. 10% anhydrous sodium sulfate solution with a volume equivalent to half of the petroleum ether layer was added to the remaining petroleum ether solution for washing up until the petroleum ether solution became neutral. At the end, the petroleum ether solution was dehydrated through anhydrous sodium sulfate, concentrated and suspended in 10 mL volume for DDT measurement.

### 2.6. Gas Chromatography Analysis of DDT

The DDT standard solution contained components of p,p′-DDE, o,p′-DDT, p,p′-DDD and p,p′-DDT, in concentrations of 20, 20, 60.08, 60 mg/L, respectively. Chromatographic grade acetone was used to dilute the DDT solution to make four concentration levels for each component. The gas chromatographic instrument used was a HP5890. Column HP-5, 30 m × 0.320 mm (id) × 0.25 μm. High purity nitrogen (99.999%) was used as the carrier gas. Gasification temperature was 220 °C, column temperature was 195°C, and detector temperature (ECD) was 245 °C. The speed of gas flow was 2 mL/min. Splitless injections (2 μL) were made. 2,4,5,6-Tetrachloro-*m*-xylene was used as the recovery indicator to control the recovery rate during the whole operation procedure. The recovery standard was used to control the sample recovery rate. The recovery rate of the indicator ranged between 78% and 88.6%. The recovery rate of DDT ranged between 88.4% and 98.7%. The detection limit for p,p′-DDE, o,p′-DDT, p,p′-DDD and p,p′-DDT was 0.902, 3.869, 2.847l and 0.756 μg/L, respectively.

### 2.7. Statistical Analysis

One-way ANOVA analysis was performed to compare the residues of four components DDT and DDTs in soils between different treatments by using SPSS11.5 software.

## 3. Results and Discussion

### 3.1. Effects of Different Atmospheres on Remediation of DDT-contaminated Soil by Laccase

**Figure 1.**
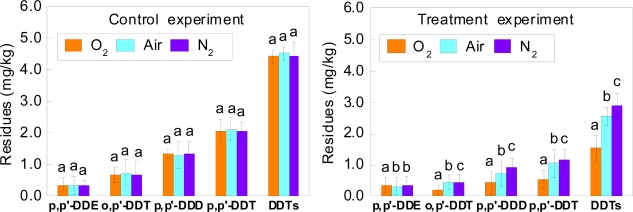
Effects of different atmospheres on degradation of four components of DDT and DDTs in soil by laccase after 25 days of incubation. Bars represent mean ± 1SE (n = 3). Bars followed by different letters are significantly different from each other at P < 0.05.

One-way ANOVA showed that the residues of the components of DDT (except for p,p′-DDE from soils with air and nitrogen) and DDTs were significantly different between each soil under different atmospheres at the end of the incubation with laccase (Figure 1). After 25 days of incubation with laccase, the reductions of residues of four components of DDT and DDTs in soils under different atmospheres are shown in Table 1.

The residues of four components of DDT and DDTs in different pH soils were not significantly different after 25 days of control experiments. The residues of DDTs in soils under different atmospheres at the end of the incubation with laccase decreased by 59.8% (O_2_), 41.4% (Air) and 32.1% (N_2_) compared with that in control experiments, respectively. It is well know that laccase (EC 1.10.3.2) uses molecular oxygen as the final electron acceptor [19], and laccase can catalyze the oxidation of substrates with concomitant reduction of molecular oxygen into water [24,25]. This study also found a positive correlation between the concentration of oxygen in soil and the degradation of DDT by laccase. The residue of DDTs in soil under the atmosphere of oxygen decreased by 28.1% compared with the atmosphere of nitrogen at the end of the incubation with laccase, suggesting that frequent plowing could promote the degradation of DDT in soil by laccase.

### 3.2. Effects of Different Flooding Conditions on Remediation of DDT-contaminated Soil by Laccase

**Figure 2.**
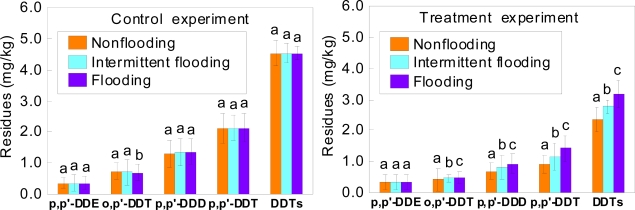
Effects of different flooding conditions on degradation of four components of DDT and DDTs in soil by laccase after 25 days of incubation. Bars represent mean ± 1SE (n = 3). Bars followed by different letters are significantly different from each other at P < 0.05.

After 25 days of incubation with laccase, the residues of the components of DDT (except for p,p′-DDE) and DDTs were significantly different between each soil with different flooding conditions (Figure 2). There were no significant effects of different flooding conditions on degradation of p,p′-DDE in soils treated with laccase (Figure 2). At the end of the incubation period, the reductions of residues of four components of DDT and DDTs in soils under flooding conditions are shown in Table 2.

The residues of the components of DDT (except for o,p′-DDT) and DDTs in soils under different flooding conditions were not significantly different after 25 days of control experiments. The residues of DDTs in soils under different flooding conditions at the end of the incubation with laccase decreased by 44.7% (nonflooding soil), 36.1% (intermittent flooding soil) and 28.0% (flooding soil) compared with that in control experiments, respectively.

In this study, a similar pattern was observed that the higher the concentrations of oxygen in soil, the lower the residues of four DDT components and DDTs in soil at the end of the experiment. The residues of four components of DDT and DDTs in the nonflooding soil declined by 2.2% (p,p′-DDE), 7.5% (o,p′-DDT), 16.8% (p,p′-DDD), 21.8% (p,p′-DDT), and 16.7% (DDTs) at the end of the incubation, compared to the flooding soil.

### 3.3. Effects of Different Soil pH on Remediation of DDT-contaminated Soil by Laccase

**Figure 3.**
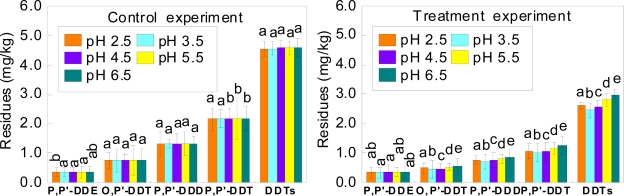
Effects of soil pH on degradation of four components of DDT and DDTs by laccase after 25 days of incubation. Bars represent mean ± 1SE (n = 3). Bars followed by different letters are significantly different from each other at P < 0.05.

In comparison, it was found out that the residues of the DDT components, except p.p′-DDT, and DDTs in soils with different pH levels were significant different at the end of the incubation with laccase (Figure 3). At the end of the incubation period, the reductions of residues of four components of DDT and DDTs in soils with different pH levels are shown in Table 3.

The residues of DDTs in soils with different pH levels at the end of the incubation with laccase decreased by 39.6% (pH 2.5), 43.4% (pH 3.5), 41.4% (pH 4.5), 36.4% (pH 5.5) and 33.0% (pH 6.5) compared with that in control experiments, respectively. The pH value can affect the activity of laccase [26]. The activity of laccase from *Polyporus sp.*, *Pleurotus sp.* and *Lentinus edodes* was the highest in a pH range of 3.2–4.5 [27]. The activity of laccase from *Polyporus sp.* was the highest at the pH of 4.2 [28]. The pH optima of fungal laccases were in the acidic pH range [19] and the stability of fungal laccase was higher at acidic pH [29]. In accordance, our results showed lower amounts of DDT in laccase treated soils at pH ranging from 2.5 to 4.5 at the end of the incubation period. The lowest amounts were found at pH 3.5.

In this study, the residues of p,p′-DDE did not show significant differences between the treatments under different soil oxygen conditions and different soil pH at the end of the incubation with laccase (Figure 1, Figure 2 and Figure 3). After 25 days of control experiments, the residues of p,p′-DDE in soils decreased by 0.1–2.6% under different atmospheres, 0.2–2.4% under different flooding conditions and 4.9–5.8% under different soil pH, respectively. Although the residues of p,p′-DDE in soils treated with laccase under different soil oxygen conditions were lower than those in control soils, the residues of p,p′-DDE in soils under different soil pH in the treatment experiments were similar with those in the control experiments. This could be explained as a two factors effect on the residual p,p′-DDE in soils under different pH. The first factor was that p,p′-DDE might be a transformation product of the laccase degradation of other components of DDT. The second one was that p,p′-DDE can also be degraded by laccase. Chu *et al*. have reported enzymes produced by plants (*Phragmites australis*), capable to degrade DDT into DDD with some DDE being formed [30]. You *et al*. found that DDE degradation was lower than DDT and DDD, due to DDE strongly bound to soil particles [31]. However, the mechanisms and pathways of the degradation of p,p′-DDE by laccase are not yet clear. Future studies will focus on exploring how the laccase degrades p,p′-DDE and DDT.

Enzymatic remediation may rapidly remove pesticide residue from the environment [18]. Enzymatic remediation of DDT-contaminated soil will be useful for the development of methods for the remediation of the contaminated soil. However, the stability of enzyme in soil may limit its application in the remediation of the polluted soil. Some new methods (e.g., immobilization of enzyme) have been developed to improve the stability of enzyme in soil [20,32].

## 4. Conclusions

The results of investigation showed that soil oxygen conditions and soil pH have significant effects on the remediation of DDT-contaminated soil by laccase. This study found a positive correlation between the concentration of oxygen in soil and the degradation of DDT by laccase. The residues of DDTs in soils treated with laccase were lower at pH range 2.5–4.5.

## Figures and Tables

**Table 1.** Reductions of residues of four components of DDT and DDTs in soils under different atmospheres at the end of the incubation period (relative to initial concentrations).

**Atmosphere**	**p,p′- DDE**	**o,p′-DDT**	**p,p′-DDD**	**p,p′-DDT**	**DDTs**
**N_2_**	1.5%	38.8%	34.7%	50.5%	40.5%
**Air**	5.4%	41.5%	48.9%	55.0%	48.5%
**O_2_**	1.9%	74.1%	68.9%	76.5%	68.6%

**Table 2.** Reductions of residues of four components of DDT and DDTs in soils under different flooding conditions at the end of the incubation period (relative to initial concentrations).

**Flooding condition**	**p,p′- DDE**	**o,p′-DDT**	**p,p′-DDD**	**p,p′-DDT**	**DDTs**
**Flooding**	3.5%	38.0%	34.1%	39.0%	34.8%
**Intermittent flooding**	3.9%	39.4%	42.3%	50.0%	42.8%
**Nonflooding**	5.7%	45.4%	50.9%	60.8%	51.5%

**Table 3.** Reductions of residues of four components of DDT and DDTs in soils with different pH levels at the end of the incubation period (relative to initial concentrations).

**Soil pH**	**p,p′- DDE**	**o,p′-DDT**	**p,p′-DDD**	**p,p′-DDT**	**DDTs**
**2.5**	5.5%	38.6%	47.0%	54.2%	46.1%
**3.5**	5.8%	42.9%	50.7%	57.9%	49.7%
**4.5**	5.7%	40.3%	48.3%	55.6%	47.4%
**5.5**	5.1%	33.4%	42.6%	50.5%	42.2%
**6.5**	4.9%	30.4%	39.5%	46.1%	38.7%
